# Active 3D Imaging of Vegetation Based on Multi-Wavelength Fluorescence LiDAR

**DOI:** 10.3390/s20030935

**Published:** 2020-02-10

**Authors:** Xingmin Zhao, Shuo Shi, Jian Yang, Wei Gong, Jia Sun, Biwu Chen, Kuanghui Guo, Bowen Chen

**Affiliations:** 1 State Key Laboratory of Information Engineering in Surveying, Mapping and Remote Sensing, Wuhan University, Wuhan 430079, China; zhaoxingmin@whu.edu.cn (X.Z.); weigong@whu.edu.cn (W.G.); cbw_think@whu.edu.cn (B.C.); kuanghuiguo@whu.edu.cn (K.G.); chenbowen1204@whu.edu.cn (B.C.); 2 Faculty of Information Engineering, China University of Geosciences, Wuhan 430074, China; yangjian@cug.edu.cn (J.Y.); sunjia@cug.edu.cn (J.S.)

**Keywords:** fluorescence LiDAR, laser-induced fluorescence, vegetation monitoring, classification discrimination

## Abstract

Comprehensive and accurate vegetation monitoring is required in forestry and agricultural applications. The optical remote sensing method could be a solution. However, the traditional light detection and ranging (LiDAR) scans a surface to create point clouds and provide only 3D-state information. Active laser-induced fluorescence (LIF) only measures the photosynthesis and biochemical status of vegetation and lacks information about spatial structures. In this work, we present a new Multi-Wavelength Fluorescence LiDAR (MWFL) system. The system extended the multi-channel fluorescence detection of LIF on the basis of the LiDAR scanning and ranging mechanism. Based on the principle prototype of the MWFL system, we carried out vegetation-monitoring experiments in the laboratory. The results showed that MWFL simultaneously acquires the 3D spatial structure and physiological states for precision vegetation monitoring. Laboratory experiments on interior scenes verified the system’s performance. Fluorescence point cloud classification results were evaluated at four wavelengths and by comparing them with normal vectors, to assess the MWFL system capabilities. The overall classification accuracy and Kappa coefficient increased from 70.7% and 0.17 at the single wavelength to 88.9% and 0.75 at four wavelengths. The overall classification accuracy and Kappa coefficient improved from 76.2% and 0.29 at the normal vectors to 92.5% and 0.84 at the normal vectors with four wavelengths. The study demonstrated that active 3D fluorescence imaging of vegetation based on the MWFL system has a great application potential in the field of remote sensing detection and vegetation monitoring.

## 1. Introduction

Plants play a considerable role in the carbon and water cycles of the global ecosystem [1,2]. A prompt and effective monitoring of vegetation is of great significance for ecological environmental monitoring and agricultural guidance. Researchers have regarded the optical remote sensing monitoring method as an ideal and feasible way, owing to its several advantages, such as quickness, accuracy, and non-destruction of plants [3]. Many optical remote sensing imaging techniques have been applied to vegetation detection in recent decades. Passive hyperspectral reflection imaging, a commonly used form of optical imaging, can provide abundant biochemical components of plants. However, such a method lacks the spatial expression in 3D space and can be affected by various factors, such as the external environment, including weather conditions, and measurement time [4,5]. The LiDAR technology, an active detection sensor with several technical advantages, such as high temporal–spatial resolution and non-destruction, has received great attention from researchers [6]. Such technology has obtained a wide range of applications on vegetation structural parameter inversion [7,8]. However, this technology typically utilizes a single band of near-infrared laser for the detection of spatial location [9], and it lacks spectral information associated with the materialized components. Therefore, the signal acquisition of multi-wavelength channels can be extended on the basis of the single-wavelength LiDAR, to expand the detection capability of vegetation biochemistry and growth state.

The fluorescence spectral properties of vegetation provide an available indicator for its detection. Fluorescence is treated as the radiation appearance of the energy loss by the oscillating motion of electrons during electromagnetic radiation [10]. The present research means for the vegetation fluorescence emission include stimulation chlorophyll fluorescence by passive solar energy and active artificial light source-laser for induction. Sun-induced fluorescence (SIF)-based vegetation remote sensing monitoring has also achieved development in recent years. SIF can provide global-scale chlorophyll fluorescence detection through on-board data and an indicator for studying the vegetation’s ecological environment [11]. However, SIF only provides the fluorescence bands (685 and 740 nm) associated with the chlorophyll in vegetation, due to the extraction method limitation [12,13,14]. This passive fluorescence detection provides fluorescence spatial distribution in 2D images only and lacks the 3D structural detection capacity of LiDAR.

Laser-induced fluorescence (LIF) is an active means of generating fluorescence by using a single short-wavelength laser as the excitation light. Vegetation absorbs the light energy of a given wavelength, and a part of it is dissipated by light emission at long wavelengths within a short time [15,16]. Vegetation has typical characteristic signals and spectral shapes in the case of being produced by laser stimulation. With the discovery and exploration of the LIF technology, monitoring vegetation by using this mechanism has become possible. Chappelle et al. [16] used an ultraviolet (UV) laser to stimulate fluorescence signals on leaves and proposed the utilization of fluorescence to distinguish vegetation types. They further explored the ability of fluorescence as a probe to resolve plant species and stress states [17]. The production of chlorophyll fluorescence peaks in vivo was also explained [18]. Researchers studied the utilization of fluorescence characteristic peaks to develop a series of correlation studies on vegetation growth status [19], biochemical content, and environmental stress factors [20]. Fluorescence characteristics have a strong indication of leaf nitrogen content [21], water deficiency [22], and fungal infections [23]. Spectral properties of LIF demonstrate a powerful ability to monitor the vegetation status as the reflection [24,25]. Artificial-light-source-induced fluorescence signals have more comprehensive spectral characteristics than SIF and are excited with only a single-wavelength light economically. Accordingly, we supposed that the LiDAR laser source is used to simultaneously achieve laser scanning ranging and fluorescence induction forming multi-wavelength reception for implementing a multi-wavelength fluorescence LiDAR. The system expands the ability to monitor the physiological states of vegetation by adding several channels for receiving fluorescence signals.

Some existing fluorescence LiDAR systems have achieved a good ability of detecting marine oil spills [26,27] and terrestrial water bodies [28]. A number of fluorescence imaging systems had been previously constructed to express the distribution characteristics of fluorescence signals [29,30]. These proactive vegetation fluorescence imaging systems can monitor growth and stress status by using the LIF technology from an imaging perspective [31]. However, these fluorescence imaging systems express the spatial distribution of fluorescence emission signals in the form of 2D images. The imaging spatial scale is extremely small to be directly applied to the remote sensing of vegetation.

Simultaneous monitoring of the external appearance and internal biochemical status has a comprehensive perception for vegetation remote sensing. We stimulate the vegetation fluorescence reception for multi-wavelength channels on the basis of the scanning ranging function of single-wavelength LiDAR. In this way, 3D spatial structural and growth state information of vegetation can be simultaneously acquired. However, constructing such LiDAR with a multi-wavelength reception of fluorescence manifests several problems. First, several wavelengths need to be selected for the fluorescence emission detection. Vegetation emits fluorescence in the form of a continuous spectrum through UV laser excitation. The receiving wavelength design can represent biochemical information and reduce the hardware cost of the system. Second, the enhancement of multi-channel fluorescence data in the background signal is also a problem, since there is a large number of non-vegetation signals as non-interested targets in the system data. Third, the data of these multiple different system units must be organized and visualized. The data output comes from several units of the system. These data require an integrated expression of the 3D spatial structure and fluorescence emissions of the vegetation.

In this study, (1) the MWFL system was proposed, created, and integrated, to perform experimental verification in the laboratory. The system design of the four wavelengths corresponding to the characteristics of the vegetation fluorescence, system components, and system-based data form were introduced. (2) The system experimented fluorescence signal imaging and scanned canopy distribution of the vegetation to verify the 3D imaging ability of the system. (3) Three-dimensional fluorescence imaging based on spectral enhancement pretreatment was adopted and achieved a good effect on the experimental scenes. (4) System evaluation based on point cloud classification was applied to classify 3D fluorescence point cloud data on vegetation, to further quantitatively explain the system advantages. The ability of the MWFL system to monitor spatial and biochemical status of vegetation through 3D fluorescence imaging was demonstrated. The feasibility of the MWFL system and the efficiency of 3D fluorescence imaging for vegetation detection were assessed.

## 2. Materials and Methods

### 2.1. System Description

#### 2.1.1. Selection of Fluorescence Wavelengths

MWFL, an active remote sensing monitoring device, adds several channels to receive the vegetation fluorescence compared with the ranging LiDAR. When excited by the short-wavelength light source, the energy of vegetation fluorescence is emitted in a longer continuous wavelength range. The fluorescence-receiving wavelengths of the system design must be optimally selected to represent vegetation fluorescence characteristics and to be as few as possible, considering the system cost.

**Figure 1:**
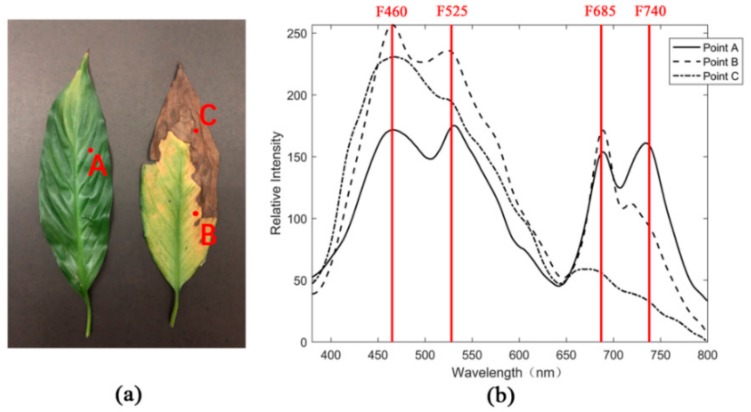
Induced continuous fluorescence spectrum of leaves in different physiological states excited with an UV laser. (**a**) Two leaves of different physiological states (Scene 1), and three points, namely A, B, and C, were located in fresh green, yellow, and brown areas, respectively; (**b**) continuous fluorescence spectrum of points A, B, and C excited with a UV laser (355 nm) was detected by the wavelength of 380–800 nm.

In this study, two leaves in different physiological states were picked. The continuous fluorescence spectra of the points measured through ICCD (Intensified Charge Coupled Device) excited with a UV laser in the laboratory were recorded. Figure 1a shows that the two leaves had different physiological states. The upper right corner of the right leaf had turned brown. Three points, namely A, B, and C, in the two leaves were located in the fresh green, yellow, and brown areas, respectively. The color characterization of the exterior leaves reflected the concentration distribution of the internal pigment. Figure 1b shows the continuous fluorescence spectral shape of points A, B, and C (wavelength range of 360–800 nm).

Vegetation has typical fluorescence spectral emission waveforms during UV laser induction and exhibits characteristic peaks, namely F460, F525, F685, and F740. The summit of F525 is sometimes less evident, or merely a slight rise on the fluorescence spectrum is observed [32]. The characteristic peaks of F685 and F740 are closely related to the chlorophyll content of leaves [33]. F460 is mainly caused by water-soluble compound NADPH, vitamin K, and beta-carotene; the prime contributor to the characteristic peak of F525 is riboflavin [32]. The measured points of the selected leaves show typical fluorescence spectral curves, but they differ from each other. Points A, B, and C represent the process of leaves turning from green to yellow and are eventually withered. Figure 1b demonstrates that the fluorescence spectrum reflects the changes in biochemical substances inside the leaves during this process. In the green leaf, chlorophyll closely related to photosynthesis reactions actively works, as indicated by F685 and F740 on the spectrum of point A. The spectrum of point B shows that the intensity of F740 first decreases, and that of F685 slightly increases when the leaf turns yellow. By contrast, F460 related to lutein and carotene can have a relatively large increase in strength. The chlorophyll content decrease is accompanied by a decrease in the F740 intensity; an increase in F685 may be due to the weak resorption effect [34]. As illustrated in Figure 1, the fluorescence spectrum of point C indicates that the strength of F460 and F525 is low when the leaves are withered, given that the corresponding biochemical substances are decreasing. Simultaneously, chlorophyll is almost exhausted, and the corresponding strength of F685 and F740 has become low, although not obvious. The change process of the fluorescence spectrum demonstrates that the intensity variation of the four characteristic wavelengths, namely F460, F525, F685, and F740, can represent the degree of yellowing in the leaves.

The developed active laser fluorescence imaging system predecessor was applied to the vegetation detection. The wavelengths of fluorescence imaging system Lichtenthaler et al. studied were blue, green, red, and far-red, corresponding to fluorescence emission [35]. Langsdorf et al. developed multicolor fluorescence imaging to determine whether the nitrogen-stress state of leaves is related to these four wavelengths [29]. The range of detection bands of fluorescence imaging systems for vegetation nutrition stress and disease diagnosis detection has been focusing on the four wavelengths, namely F460, F525, F685, and F740 [31,36,37], in recent years, in spite of a slight offset in the wavelength position. Considering the point measurement results of the vegetation fluorescence spectrum in the laboratory and receiving bands of the previous fluorescence imaging system, 460, 525, 685, and 740 nm were selected as the four receiving wavelength centers of the MWFL system.

#### 2.1.2. System Components

**Figure 2:**
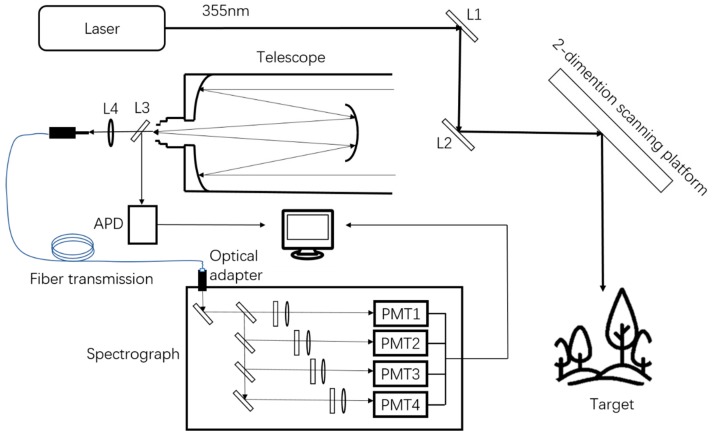
Block diagram of the multi-wavelength fluorescence LiDAR.

The MWFL system design aims to simultaneously obtain the 3D spatial structure and four wavelengths of the fluorescence characteristics of the vegetation target. This system can implement two detection mechanisms, namely reflection ranging and laser-induced fluorescence. In addition to the scanning and ranging functions of the single-wavelength LiDAR, the system also has the module for fluorescence detection and reception. The MWFL system includes system components of laser emission, scanning, ranging, receiving detection, and data processing. Figure 2 shows the block diagram of the MWFL system.

In the MWFL system, the laser source uses a 355 nm UV laser as a laser-emitting unit considering excitation efficiency. The UV laser is not only the excitation source of vegetation fluorescence, but its reflective signal is the system’s distance-measuring source. The parameters of the laser source are set to meet the requirements of laser pulse ranging and vegetation fluorescence induction. The L1 mirror has high reflectivity to the UV-wavelength laser, which acts as a reflection and filter, for optimal design. The L2 reflective mirror reflects the laser light to the center of the 2D scanning platform of a scanning unit. The beam can be scanned in the x and y directions on vegetation canopy target as the platform rotates. The system echo signals, including reflective UV laser and vegetation fluorescence signals, are received through the view field of Schmidt–Cassegrain telescope. The connection between the center of the scanning platform and the center of the L2 mirror is collinear with the central axis of the telescope to form an optical coaxial design. Such a setup is the requirement for ranging and spectral detection in the single point and is beneficial to improve the detection signal to noise ratio.

The receiving detection unit mainly includes objects, such as telescope, spectrometer, and transmission fiber. The L3 mirror can reflect the UV band and transmit the long band, which can separate the UV reflection and fluorescence signal from vegetation. The reflective signal is recorded by an APD (Avalanche Photo Diode) of the ranging unit, which is compared with the time of the initial pulse by means of TOF (Time of Flight), to obtain the distance value of the single point through the pulse method. After focusing through the L4 convex lens and coupling, fluorescence signals are transmitted through the fiber to the spectrometer. Inside the spectrometer are a four-wavelength splitter module and corresponding photodetectors. With regard to the spectroscopic module, the continuum signal of the vegetation fluorescence introduced into the spectrometer is separated from each other by dichroic filters passing through narrow-band filters and into the four photomultiplier tube arrays with single-wavelength response centers on 460, 525, 685, and 740 nm. The four-channel photoelectric signal is converted by analog–digital transformation. The fluorescence intensity is acquired by integration and transmitted to the data-processing unit of the system. Table 1 shows the technical parameters of MWFL system.

Compared with the existing vegetation-monitoring fluorescence LiDAR [38,39], the MWFL system has a combination of scanning ranging and LIF to achieve 3D fluorescence imaging of vegetation targets. This imaging method can form an integrated monitoring of the vegetation’s external growth and internal biochemical components.

#### 2.1.3. Data Description

**Figure 3:**
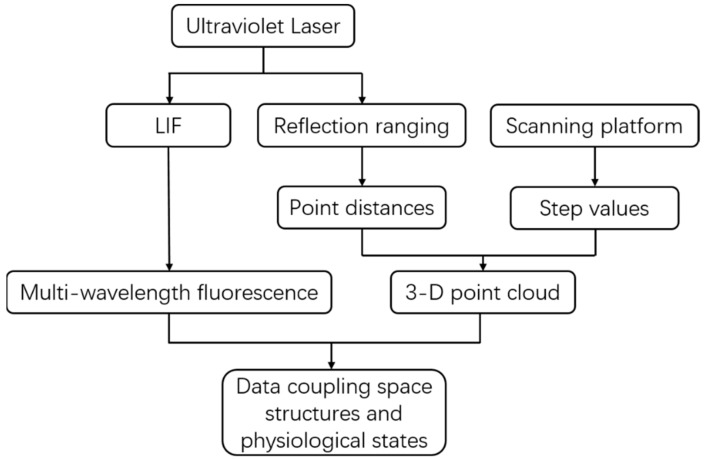
Data formation process of multi-wavelength fluorescence LiDAR.

The form of the MWFL system data is spatially presented in a point cloud format. Each point has a fluorescence spectral property. The MWFL system breaks through the limitations of traditional single-wavelength ranging LiDAR only for 3D space detection, given its fluorescence spectral features and expanded ability to detect vegetation. The type of system data is divided into two parts: 3D point cloud data and four-wavelength data of fluorescence signal. Figure 3 illustrates the formation process of the MWFL system data form.

Figure 3 shows that the UV laser generates the multi-wavelength fluorescence signal for a single point via LIF, and its reflection is used for ranging. The design method for single-point measurement was described in Section 2.1.2. The data-processing unit of the MWFL system records the distance values at the single-point position and the fluorescence intensity values of the four channels. The system scanning platform can be rotated in two directions, to perform 2D scanning detection on vegetation targets. The rotary step values are simultaneously recorded. The data that are saved and transferred to the data-processing unit consisted of three parts: the distance values of points, the signal intensity of four channels, and the step values of the platform scanning. Among these parts, the distances and step values between points constitute the 3D point cloud spatial distribution in the form of spherical coordinates. These coordinates can be converted into a spatial Cartesian coordinate system. The vegetation fluorescence intensity in the four channels constitutes multi-wavelength fluorescence characteristic data. The data outputted by the system include the XYZ coordinates and the fluorescence intensity values of F460, F525, F685, and F740 of the vegetation target. The system generates a remote-sensing data form for vegetation targets. This new form of data couples the 3D distribution and fluorescence spectra of vegetation detection. As a result, the integrated monitoring of spatial and physiological status of vegetation target is enabled. We hope that this new data format based on the MWFL system can be applied to the remote-sensing monitoring of vegetation for improving the accuracy of qualitative and quantitative detections of vegetation.

### 2.2. Sample Materials

Two scenes were presented as samples to demonstrate the ability of MWFL system’s 3D fluorescence imaging in characterizing the vegetation states and the ability to couple spatial and physiological states. The two leaves mentioned in Section 2.1.1 for wavelength selection were recommended as Scene 1 to implement 3D fluorescence imaging on the basis of point cloud. This task was carried out to study the spectral-imaging differences in the green, yellow, and brown areas of the leaf.

**Figure 4:**
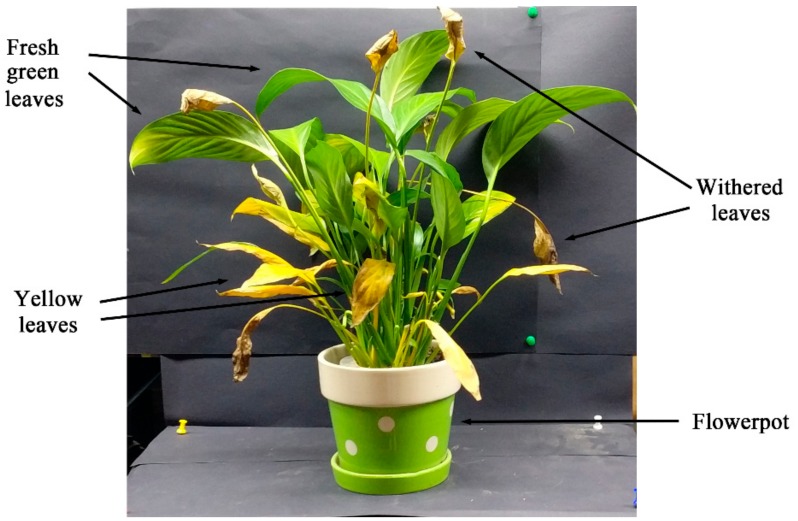
Scene 2 for 3D point cloud imaging of the MWFL scanning experiment.

A scanning experiment of the potted vegetation was conducted, to prove the detection advantage of the system on the 3D canopy as Scene 2 in an experimental scene (Figure 4). The leaves in this potted vegetation were spatially distributed at different angles and positions. Moreover, the leaves represent their different physiological states. Such a featured scene can be used as an observation sample with spatially complex states and physiological differences. For two scenes arranged in the laboratory, the ability of MWFL system to effectively monitor vegetation can be verified. Scene 1 expresses the spectral detection performance of the system for fluorescence emission at the leaf level. Scene 2 shows the fluorescence point cloud imaging capability of vegetation with 3D morphology.

### 2.3. Methods

#### 2.3.1. D Fluorescence Imaging Based on Spectral Enhancement

The spectral signal of the MWFL system comes from the photoelectric conversion of four channels. However, the fluorescence spectral information of vegetation from the target of interest is often insufficiently prominent, due to the ground-scene background. During the 3D imaging of vegetation fluorescence, appropriate methods should be adopted to highlight the fluorescence characteristics of vegetation for adapting to the perception of human eyes. The method of processing remote-sensing hyperspectral image uses hyperspectral enhancement application and obtains exceptional analytical results [40,41]. Histogram equalization (HE) is a commonly used image spectral enhancement method that redistributes the spectra intensity by histogram distribution [42]. In this work, the raw spectral data obtained by the system were processed by the HE method. The fluorescence characteristics of the vegetation point cloud after treatment in this way were significantly and visually enhanced. The signal strength pseudo-color imaging of point cloud in four wavelengths can represent spatial changes in leaves in different physiological states.

#### 2.3.2. System Evaluation Based on Point Cloud Classification

The MWFL system expands the detection capability of the physiology and growth status through the LIF mechanism for traditional LiDAR. The four added bands multiply the amount of information contained in the system data compared with the single-wavelength LiDAR. The improvement of vegetation-recognition ability via the increased four-wavelength fluorescence must be quantitatively evaluated. In this study, the point cloud with multichannel fluorescence properties was analyzed by classifying the different conditions of the leaves. The point cloud classification analysis included the classification of data within four channels and the comparative classification of the spatial parameter and four channels with that.

Support vector machine (SVM), which is a popular machine learning method, has been widely applied for data classification and regression [43,44]. SVM has certain advantages, such as robustness and demanding small sample size of remote sensing data for training [45]. Such a method is adaptive for classifying the spatial and spectral feature data of the system. This method was used for point cloud classification, to demonstrate the effectiveness of the 3D fluorescence data of the system for vegetation detection.

The classification for system data is for Scene 2 because the data of Scene 1 are the representation of fluorescence detection in a planar form on the MWFL system. The single-, double-, and four-wavelength spectral data from Scene 2 were used as input eigenvalues of the model classifier for classification and analysis. The normal vector is a commonly used parameter and is related to the spatial structure in vegetation detection [46]. The normal vectors of the point cloud were used to indicate the recognition ability of the single-wavelength LiDAR. The classification results of the fluorescence data of four wavelengths with normal vectors were compared. In the training process of SVM classification, due to the difference in the sample sizes of ground categories, the training samples were selected within a category in turn. The classification selected 2-fold cross-validation—that is, 50% training and 50% testing—and SVM kernel function choose the linear.

Moreover, the overall classification accuracy of the point cloud results can be affected by the imbalance of the sample size of each category [47]. The Kappa coefficient [48] was also used as a parameter to evaluate the overall classification in combination with the classification accuracy.

## 3. Results

For 3D imaging of fluorescence, the space point cloud is formed by reflective ranging of the ultraviolet light and the rotary step values of the scanning. The point cloud data include the distance values obtained by TOF method and the step values. In the experiments of Scene 1 and Scene 2, the detection distance is about 3.5 m, and the point–point distance is about 3.5 mm. The space size of Scene 1 is 0.12 m × 0.22 m, and Scene 2 is 0.25 m × 0.28 m.

**Figure 5:**
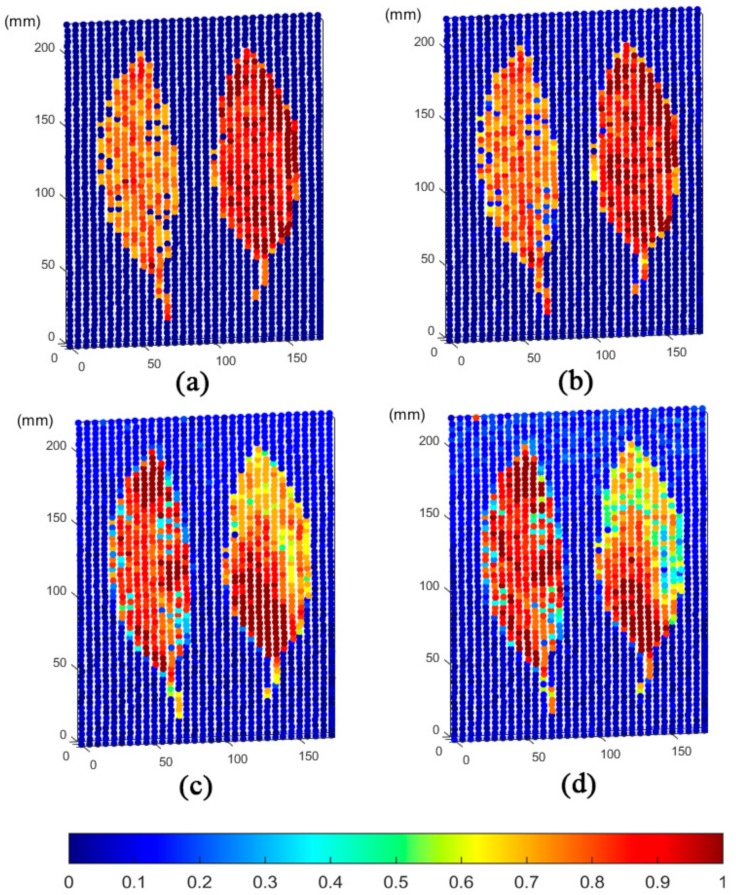
Single-wavelength fluorescence 3D imaging based on scene 1 scanning experiment of the MWFL system: (**a**) 460 nm, (**b**) 525 nm, (**c**) 685 nm, and (**d**) 740 nm wavelength signal intensity pseudo-color 3D imaging. The intensity values were subjected to histogram equalization (HE) and normalization.

Figure 5 shows the 3D imaging result of the fluorescence point cloud of Scene 1. The results of point cloud imaging showed the characterization of the vegetation’s physiological states by the four-channel fluorescence signal, given that Scene 1 landed the leaves on the blackboard.

The imaging results of Figure 5 demonstrate that the leaf and non-leaf backgrounds are displayed in the form of point cloud and show significant differences. In the fresh green, yellow, and brown areas of leaves, the four-wavelength spectral point cloud imaging presents visual features that match themselves. However, the left and right leaves in Scene 1 exhibit significant differences in these four characteristic wavelengths. The leaf on the left represents the green state of the vegetation. The intensity values at 460 and 525 nm wavelengths are significantly lower than the yellow and brown-leaf regions on the right. The brown area of the right leaf exhibits an extremely high intensity at 460 and 525 nm wavelengths due to the increased degree of the yellowing of the leaves. Fluorescence intensity values at 685 and 740 nm are correlated in most regions of point cloud. The green area of the left leaf and the yellow area of the right leaf are stronger than the brown area because the chlorophyll content in the latter was exhausted. The tip portion of the upper side of the green leaf on the left has similar intensity values, at 685 and 740 nm, to the high intensity of the yellow region. The realistic picture of Scene 1 shows that the green color in this area was declining. The actual colors between the two areas are similar. The high-intensity values of most green areas might be due to the decrease of water content or the nonlinear relationship between chlorophyll fluorescence intensity and chlorophyll content. In the portion where the yellow and brown areas on the right side of the right leaf are bordered, that is, the position of point B at wavelength selection in Section 2.1.1, the fluorescence intensity at 685 nm is slightly stronger than that at 740 nm. Such an outcome is consistent with the test result of the continuous spectrum on point B. This finding indicates that a change buffer distribution of the internal physiological state occurs between the yellow and brown areas of the leaf. The spatial distribution change was revealed by fluorescence imaging of the MWFL system.

**Figure 6:**
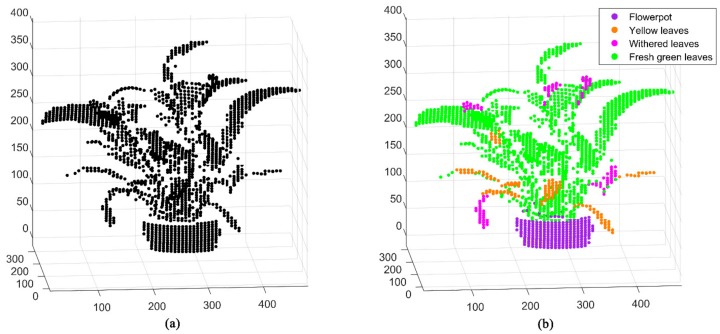
Scene 2 scanning experiment 3D point cloud based on MWFL system. (**a**) Three-dimensional distribution of spatial point cloud; (**b**) The ground truth categories given for classification.

Experiments on the spatial distribution of Scene 2 based on MWFL system are performed (Figure 6a). The spatial 3D geometry distribution of the potted vegetation in Figure 4 is presented by the MWFL system. The manual labels were given as real categories of Scene 2, in preparation for classification (Figure 6b). In comparison with Figure 4, Scene 2 was divided into four categories: flowerpot, yellow leaves, withered leaves, and fresh green leaves (Figure 6b).

**Figure 7:**
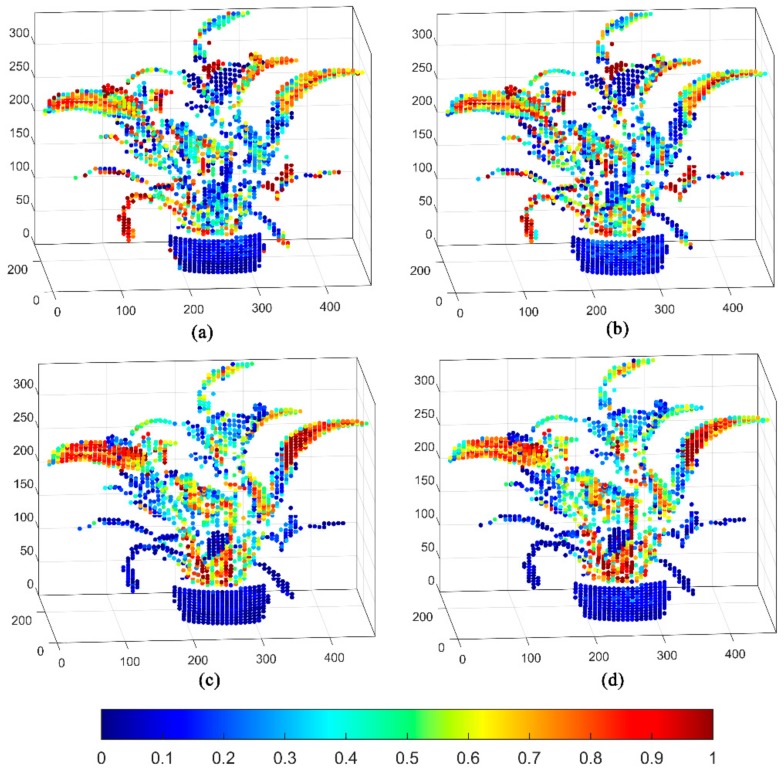
Single-wavelength fluorescence 3D imaging based on the Scene 2 scanning experiment of the MWFL system: (**a**) 460 nm, (**b**) 525 nm, (**c**) 685 nm, and (**d**) 740 nm wavelength signal intensity pseudo-color 3D imaging. The intensity values were subjected to HE and normalization.

Figure 7 displays the four-wavelength 3D spectral intensity imaging results of Scene 2. Four single-wavelength signal intensity pseudo-color 3D imaging interpretations are presented. In the point cloud imaging of Scene 2, the imaging results of the four channels show an excellent spatial distribution and spectral detection capability. The flowerpot exhibited low values in the four-wavelength 3D fluorescence intensity imaging. Green leaves exhibit high intensity at 685 and 740 nm wavelengths. Such leaves also present weak fluorescence signals at 460 and 525 nm wavelengths. Meanwhile, the withered leaves in this scene show high intensity at 460 and 525 nm wavelengths. The signals at 685 and 740 nm wavelengths are barely high. However, the yellow leaves exhibited low intensity in four channels. After that, we classified and analyzed the fluorescence point cloud data obtained by the MWFL system. This step was conducted to quantitatively estimate and describe the recognition ability of vegetation in Scene 2.

## 4. Discussion

The four single-wavelength spectral signal intensity imaging on the potted vegetation of Scene 2 demonstrated the spatial and spectral combined imaging potential of the MWFL system. The spatial variation distribution of the leaf spectrum in Scene 2 showed the continuous change of the spectrum in space to a certain extent.

**Figure 8:**
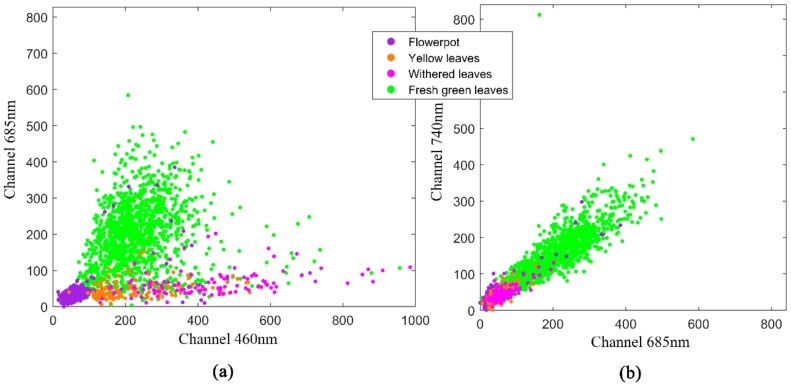
Scatter distribution of the channels’ intensity of the ground categories in scanning experiment of Scene 2: (**a**) Channels 460 nm vs. 685 nm; (**b**) Channels 685 nm vs. 740 nm.

Due to the influence of the spatial-distribution conditions, the fluorescence intensity can be affected by factors such as the distance and angle of the system observation [49]. In addition, the laser echo has the mixtures of vegetation and background targets during the scanning experiment. These factors all affect the expression of fluorescence in 3D point cloud. Figure 8 shows the original signal-intensity distribution of different ground categories in Scene 2. As shown in Figure 8a, it is clear that green leaves exhibit high chlorophyll fluorescence intensity, and flowerpots, as non-plant targets, have almost no signal in these two wavelengths. Yellow leaves in Scene 2 have almost no chlorophyll fluorescence, with some fluorescence emission in 460 nm wavelength. Compared with yellow leaves, withered leaves have a significantly enhanced fluorescence emission at 460 nm. From Figure 8b, the correlation between the intensity of two chlorophyll fluorescence bands 685 and 740 nm in Scene 2 is high. However, the difference between these two bands can be reflected in the imaging of Scene 1. The distribution of the intensity of the features in Scene 2 in the channel shows the separability of the data, which means the possibility of the classification.

**Figure 9:**
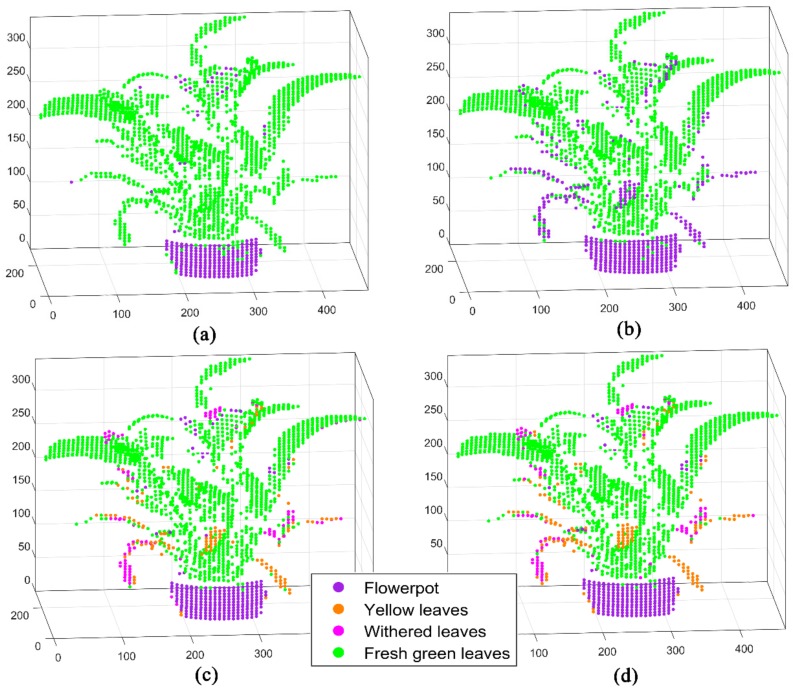
Different wavelength spectral data classification result graphs based on the MWFL system scanning in Scene 2. (**a**) Single-wavelength (460 nm); (**b**) Single-wavelength (685 nm); (**c**) Double-wavelength (460 nm + 685 nm); (**d**) Four-wavelength intensity data for classification.

The effectiveness of the data obtained by the four added channels, especially the fluorescence data, was evaluated by point cloud classification. SVM acted as a classifier to distinguish the categories of Scene 2. We presented the classification results of two single-wavelengths (460 and 685 nm), double-wavelength combination (460 nm + 685 nm), and four wavelengths as input eigenvalues (Figure 9). The classification accuracies of the four single-wavelength were basically similar. Therefore, two of four single-wavelength were representatively displayed as minimum and maximum classification accuracies. The results of the double-wavelength combination classification were equal; hence, only one combination was selected. Table 2 shows the confusion matrix of Figure 9a–d.

Figure 9 and Table 2 demonstrate that the species-recognition accuracy is gradually increasing from single to double to four wavelengths. Such accuracy was limited in the case where only single-wavelength data were applied. The overall accuracies of the single-wavelength classifications are 70.7% and 76.2%. Such a result is attributed to the simple category division, and the number of fresh green leaves account for a large proportion, thereby resulting in a high classification. However, the confusion matrices demonstrate that the single-wavelength data have almost no ability to distinguish between yellow and withered leaves of the vegetation. The Kappa coefficients of the two single-wavelength classifications also illustrate that. The classification to the kappa values of 0.17 and 0.43 were not ideal. If the double-wavelength data were used for classification, then the recognition ability would be significantly improved. The classification accuracy of this case is 81.3%, and the Kappa coefficient increased to 0.56, reflecting an improvement compared with those of single-wavelength data. The result of the four-wavelength classification reveals that the classification accuracy reaches 88.9%, and the Kappa coefficient increases to 0.75. Such a finding indicates that the classification results (see Figure 9d) are consistent. The application of four-wavelength data further improves the identification capability of vegetation physiological states. This finding illustrates the necessity for four wavelengths to detect vegetation fluorescence. From the classification of the flowerpot, the system also has a certain degree of detection ability for the background objects during vegetation-detection fieldwork.

**Figure 10:**
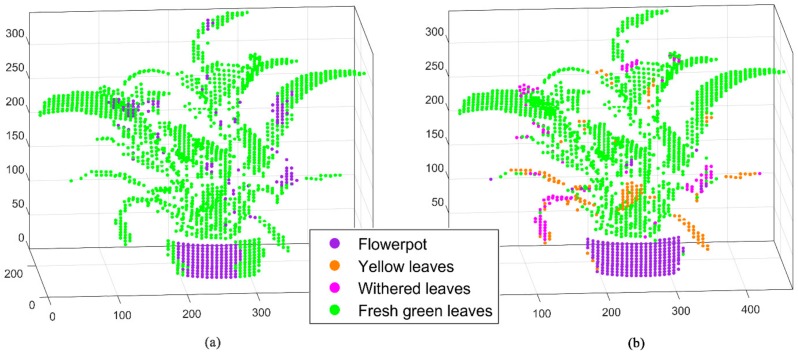
Normal vectors and normal vectors with four-channel spectral classification results (Scene 2): (**a**) Normal vectors data and (**b**) Normal vectors with four-channel spectral data for classification.

The normal vector was used as the representative parameter of the spatial structural state to be classified (Figure 10a). The normal vectors were computed by searching the neighbor points of the single point on the basis of the KNN algorithm and calculating the vertical pointing of the fitted plane. The normal vectors and four-wavelength signal values were used together for classification (Figure 10b). Table 3 shows the confusion matrix diagrams of Figure 10a,b.

The results in Figure 10 and Table 3 illustrate that the spatial parameter normal vectors present a very unsatisfactory classification outcome for the complex structure of vegetation (classification accuracy 76.2% and Kappa coefficient 0.29). In terms of the point cloud of Scene 2, the spatial shapes and angles of vegetation leaves vary, and certain leaves have few points. Figure 10a demonstrates that the normal vectors can distinguish a part of the flowerpot and most fresh green leaves, which are also related to a large number of the fresh green leaves’ points. The normal vectors for yellow and withered leaves are completely indistinguishable. However, the ability of fluorescence to indicate the physiological state are exerted when the normal vectors and the four-wavelength spectral data are combined as multi-eigenvalues (Figure 9b). The classification accuracy improves to 92.5% with the significant enhancement of the producer, user, and overall accuracies. The kappa coefficient increases to 0.84, which is a promotion relative to the four-wavelength classification.

From the results of classification, the detection of vegetation fluorescence was improved to a higher level compared to the space detection capability of the single-wavelength LiDAR. Therefore, the four-wavelength signals detected by the MWFL system can effectively improve the recognition ability of different growth states of vegetation through the LIF mechanism. Such a mechanism effectively works with spatial parameters, which single-wavelength LiDAR possesses. The coupled detection of these two mechanisms has great potential for the remote-sensing field.

## 5. Conclusions

The proposed MWFL system expands the fluorescence characteristic generated by the LIF in four wavelengths, on the basis of the ranging LiDAR. We believe that the four-wavelength detectors added to the system could represent the internal components of the vegetation. The data form of the MWFL system can be coupled with the 3D spatial structural state and the physiological state information of vegetation monitoring through data organization. The combination of two mechanisms enhances the ability to identify and monitor vegetation targets. The significance of 3D fluorescence imaging of vegetation is that it not only expresses the growth status from the outer space, but also expresses the stress status of the internal physiological status. For different types of vegetation, in addition to the different spatial-expansion states of external growth, the internal biochemical content also varies greatly. The ability of fluorescence features to qualitatively and quantitatively indicate vegetation has improved the capability of LiDAR monitoring. This monitoring method is of great benefit to forestry development and precision agriculture.

At present, the signals of the 460 and 525 nm wavelengths have a relatively high correlation. The necessity for designing these two bands may be performed by the quantitative monitoring of the vegetation in the future.

Our analysis reflects the effectiveness of the 3D fluorescence imaging monitoring on the basis of the MWFL system for vegetation remote sensing. The technical upgrades and performance optimization of the system are required if there are platform operations and large space–time scale applications. In the MWFL system, the radiation correction of distance and angular polarization are beneficial for the quantitative monitoring of the surface vegetation.

## Figures and Tables

**Table 1:** Technical parameters of MWFL system design.

Multi-Wavelength Fluorescence LiDAR	
Laser wavelength	355 nm
Repetition rate	7 kHz
Pulse width	3~5 ns
Pulse energy	18 μJ
Beam divergence	<1 mrad
Telescope aperture	200 mm
Spatial resolution	Distance: 10 mm
	Scanning: 2 mm @20m

**Table 2:** Confusion matrices of different wavelengths’ spectrum data classification results for Scene 2 (corresponding to Figure 9).

	Ground Truth	Predicted Class				Producer Accuracy
		Flowerpot	Withered Leaves	Yellow Leaves	Fresh Green Leaves	
(a) 460 nm	Flowerpot	108	0	0	140	0.44
	Withered leaves	16	0	0	100	0
	Yellow leaves	4	0	0	129	0
	Fresh green leaves	132	0	0	1147	0.90
User accuracy		0.42	0	0	0.76	
**Overall accuracy (%): 70.7%**						
**Kappa coefficient: 0.17**						
(b) 685 nm	Flowerpot	235	0	0	13	0.95
	Withered leaves	30	5	0	81	0.04
	Yellow leaves	1	1	0	131	0
	Fresh green leaves	159	6	0	1114	0.87
User accuracy		0.55	0.42	0	0.83	
**Overall accuracy (%): 76.2%**						
**Kappa coefficient: 0.43**						
(c) 460 nm + 685 nm	Flowerpot	236	2	3	7	0.95
	Withered leaves	51	13	6	46	0.11
	Yellow leaves	5	12	18	98	0.14
	Fresh green leaves	55	22	25	1177	0.92
User accuracy		0.68	0.27	0.35	0.89	
**Overall accuracy (%): 81.3%**						
**Kappa coefficient: 0.56**						
(d) Four wavelengths	Flowerpot	240	0	4	4	0.97
	Withered leaves	7	57	19	33	0.49
	Yellow leaves	0	13	69	51	0.52
	Fresh green leaves	24	20	23	1212	0.95
User accuracy		0.89	0.63	0.60	0.93	
**Overall accuracy (%): 88.9%**						
**Kappa coefficient: 0.75**						

**Table 3:** Confusion matrices of the normal vectors and normal vectors with four-channel spectral classification results for Scene 2 (corresponding to Figure 10).

	Ground Truth	Predicted Class				Producer Accuracy
		Flowerpot	Withered Leaves	Yellow Leaves	Fresh Green Leaves	
(a) Normal vectors	Flowerpot	107	0	0	141	0.43
	Withered leaves	8	7	6	95	0.06
	Yellow leaves	3	0	9	121	0.07
	Fresh green leaves	48	0	0	1231	0.96
User accuracy		0.64	1.0	0.60	0.78	
**Overall accuracy (%): 76.2%**						
**Kappa coefficient: 0.29**						
(b) Normal vectors + four wavelengths	Flowerpot	244	0	0	4	0.98
	Withered leaves	4	73	13	26	0.63
	Yellow leaves	3	14	91	25	0.68
	Fresh green leaves	6	14	24	1235	0.97
User accuracy		0.95	0.72	0.71	0.96	
**Overall accuracy (%): 92.5%**						
**Kappa coefficient: 0.84**						
